# Sustainable Lavender Extract-Mediated Synthesis of Silver Nanoparticles and Their Use in Fabricating Antibacterial Polymer Nanocomposites

**DOI:** 10.3390/nano16020098

**Published:** 2026-01-12

**Authors:** Lívia Mačák, Oksana Velgosová, Erika Múdra, Marek Vojtko, Silvia Ondrašovičová

**Affiliations:** 1 Institute of Materials, Faculty of Materials, Metallurgy and Recycling, Technical University of Košice, Letná 9/A, 042 00 Košice, Slovakia; 2 Division of Ceramic and Non-Metallic Systems, Institute of Materials Research, Slovak Academy of Sciences, Watsonova 47, 040 01 Košice, Slovakia; emudra@saske.sk (E.M.); mvojtko@saske.sk (M.V.); 3 Department of Biology and Physiology, University of Veterinary Medicine and Pharmacy in Košice, Komenského, 040 01 Košice, Slovakia; silvia.ondrasovicova@uvlf.sk

**Keywords:** silver nanoparticles, polymer, electrospinning, nonwoven textile, nanocomposite, antibacterial properties

## Abstract

This study focuses on the development of antibacterial polymer nanocomposites based on biologically synthesized silver nanoparticles (AgNPs) and polyvinyl alcohol (PVA) as the polymer matrix. Silver nanoparticles were produced using an aqueous extract from dried *Lavandula angustifolia* (lavender) leaves, which proved to be highly effective in reducing silver ions and stabilizing the resulting nanoparticles. The synthesized AgNPs were characterized by FTIR, UV-Vis, TEM, SEM, and DLS analyses. The nanoparticles were predominantly spherical, with more than 70% having diameters below 20 nm. Subsequently, AgNPs were incorporated into the PVA matrix via an ex situ approach to fabricate nanocomposite fibers and thin films. SEM analysis confirmed successful incorporation and uniform distribution of AgNPs within the polymer structures. The nanocomposites exhibited pronounced antibacterial activity against both Gram-positive (*Staphylococcus aureus*, *Staphylococcus haemolyticus*, *Streptococcus uberis*) and Gram-negative (*Escherichia coli*, *Pseudomonas aeruginosa*) bacteria, with nanofibers demonstrating superior performance compared to thin films. These findings highlight the potential of lavender-extract-mediated AgNPs as sustainable functional fillers for the fabrication of eco-friendly antibacterial materials applicable in biomedical and food packaging fields.

## 1. Introduction

Nanotechnology is a rapidly advancing field enabling the design of materials with unique physical, chemical, and biological properties [1]. Among nanomaterials, metallic nanoparticles, particularly silver nanoparticles, have attracted significant attention due to their strong antibacterial, antiviral, and antifungal activities [2,3,4]. These properties make AgNPs valuable in medicine (e.g., wound dressings, implants, coatings), food packaging, textiles, and cosmetics.

The growing resistance of microorganisms to conventional antibiotics poses a major global health threat [5,6,7], especially in clinical and public environments, where contaminated surfaces can facilitate infection spread. This situation highlights the urgent need for new antimicrobial materials that combine high efficacy with environmental safety. In this regard, nanomaterials synthesized via green and sustainable methods are increasingly recognized as promising alternatives to traditional chemical disinfectants and antibiotics [8,9,10,11,12].

From an environmental and safety perspective, the biological synthesis of AgNPs is gaining increasing importance. This method utilizes natural extracts from plants, microorganisms, or animal-derived products as reducing and stabilizing agents. Such an approach eliminates the need for toxic chemicals and represents an eco-friendly alternative to traditional chemical synthesis methods.

*Lavandula angustifolia* leaves contain a complex mixture of secondary metabolites, including phenolic acids and flavonoids (e.g., rosmarinic-acid-type phenolics), terpenoids, and other oxygen- and nitrogen-containing biomolecules. These compounds can act as reducing agents (donating electrons to Ag^+^) and as capping/stabilizing agents by adsorbing on the nanoparticle surface via –OH, C=O, and –NH functional groups. The chemical profile of lavender is known to vary with geographic location, climate, and harvest conditions, which can influence extract composition and consequently nanoparticle formation and stability [13].

For practical applications, it is often necessary to incorporate AgNPs into a suitable matrix, which expands their potential use [14,15]. Various types of polymer matrices can be combined with different types of nanoparticles to produce nanocomposites with specific functional properties, such as antimicrobial activity, photocatalytic efficiency, or electrical conductivity [16]. By combining polymer with AgNPs, materials with enhanced properties, such as antimicrobial properties, can also be obtained [17,18]. The polymer matrix provides mechanical stability, good processability, and controlled release of nanoparticles, which is essential for creating materials with long-lasting antimicrobial protection. Polyvinyl alcohol (PVA) is one of the most used polymers due to its water solubility, biocompatibility, and ability to form homogeneous films and fibers [19,20].

Innovative and practically applicable products made using polymer matrix-silver nanoparticle nanocomposites offer new possibilities for preventing the spread of pathogens in various environments [21,22,23]. *Streptococcus uberis* is a common cause of mastitis in dairy cows, with infection transmission potentially occurring through contaminated water. For example, drinking containers surface modification using thin layers of PVA–AgNPs can significantly reduce the risk of bacterial contamination in drinking water containers, thereby contributing to the protection of livestock from infection.

Commonly occurring pathogenic bacteria such as *Staphylococcus aureus*, *Staphylococcus haemolyticus*, *Streptococcus uberis*, *Escherichia coli*, and *Pseudomonas aeruginosa* are known for their ability to colonize various surfaces and form biofilms, which makes their removal by conventional cleaning or disinfecting agents considerably more difficult. These microorganisms cause a wide range of infections from skin and respiratory tract infections to sepsis and hospital-acquired (nosocomial) infections. Therefore, it is essential to develop new surface protection materials that exhibit long-lasting antimicrobial activity while being environmentally friendly. In addition to use as antimicrobial surface coatings, these materials can be applied in the production of plastic containers and various items with broad applicability, particularly in medicine (e.g., packaging for sterile instruments, disposable medical devices) and the food industry (e.g., packaging materials with active protection against microbial spoilage).

This work aims to prepare polymer AgNPs nanocomposites with antibacterial properties. Silver nanoparticles will be prepared by biological synthesis and incorporated into a polymer matrix by an ex situ method. The resulting nanocomposites will be produced as thin films and nanofibers. These materials will be characterized and tested for antibacterial activity against selected Gram-positive and Gram-negative bacteria.

Although lavender mediated AgNPs synthesis has been previously reported [24], the novelty of this study lies in the use of an aqueous lavender leaf extract prepared under defined conditions as a dual reducing and stabilizing agent yielding long-term stable AgNPs, the ex situ incorporation of biogenic AgNPs into a PVA matrix followed by fabrication into two relevant material forms, electrospun nanofibers and cast thin films, using optimized processing conditions, and the systematic antibacterial evaluation against both Gram-positive and Gram-negative strains, including the veterinary relevant *S. uberis* and *S. haemolyticus*. This combination provides a practical route toward antibacterial nanocomposites suitable for surface protection and packaging applications.

## 2. Materials and Methods

Silver nitrate (AgNO_3_, >98%, Mikrochem Ltd., Pezinok, Slovakia) was used as the silver precursor for nanoparticle synthesis. The biological material (lavender leaves) employed for the preparation of extract was collected in the vicinity of Košice (Slovakia) during July and August. Polyvinyl alcohol (PVA; 98.0–98.8% hydrolysed, M.W. 146,000–186,000 g/mol; Mikrochem Ltd., Pezinok, Slovakia) served as the polymer matrix for the preparation of nanocomposites. For toxicity and antibacterial testing, the following bacterial strains were used, provided by the University of Veterinary Medicine and Pharmacy in Košice: *Staphylococcus haemolyticus* (*S. haemolyticus*) CCM 2737, *Staphylococcus chromogenes* (*S. chromogenes*) CCM 3386 (Ref. No. 4497), *Staphylococcus aureus* (*S. aureus*) CCM 4750, *Streptococcus uberis* (*S. uberis*) CCM 4617, and *Pseudomonas aeruginosa* (*P. aeruginosa*) CCM 1960. All strains were obtained from the Czech Collection of Microorganisms (Brno, Czech Republic).

Deionized water was used for all solution preparations, dilutions, and washing steps.

### 2.1. Preparation of Extract

Lavender leaves (400 g) were washed twice with deionized water to remove surface impurities, then dried at 45 °C. The dried biomass (2.76 g) was ground in a mortar and added to 100 mL of deionized water. The mixture was heated in a water bath at 80 °C for 10 min to extract bioactive components. The resulting solution was filtered through Filtrak 389 filter paper (Ref. No. 3.102.185; Filtrak, Wiesenbad, Germany) to remove solid residues. To ensure complete clarification, the filtrate was centrifuged at 9000 rpm for 30 min. The aqueous extract was stored at 4 °C and used within one week of preparation.

### 2.2. Biological Synthesis of Silver Nanoparticles

An aqueous solution of silver nitrate (AgNO_3_) with a concentration c_Ag_ = 500 mg/L was used as a precursor for the synthesis of silver nanoparticles. As a reducing and stabilizing agent, the extract of lavender leaves was used. The process of synthesis was as follows: the AgNO_3_ stock solution (1000 mL) was poured into an Erlenmeyer flask (Fisherbrand™ borosilicate glass Erlenmeyer flask, Fisher Scientific, Bratislava, Slovakia). The solution was heated to 80 °C under continuous stirring. Once the target temperature was reached, a precisely measured amount of the extract (100 mL) was slowly added dropwise to the flask under continuous stirring (dropwise addition took approximately 2 min). After the addition of the extract, the mixture was stirred at 80 °C for an additional 20 min. The reaction mixture was then cooled to room temperature and subsequently analysed by UV-vis spectrophotometry to confirm and characterize the AgNPs.

### 2.3. Nanocomposite Production

Polyvinyl alcohol (PVA) was used as the polymer matrix, while biologically synthesized silver nanoparticles served as the secondary phase. The incorporation of AgNPs into the matrix was performed using an ex situ approach.

The colloidal suspension of AgNPs was concentrated by centrifugation at 9000 rpm for 20 min. After centrifugation, the supernatant was decanted, and the concentrated nanoparticle sediment was collected from the bottom of the centrifuge tubes.

PVA powder was dissolved in deionized water under continuous stirring at 600 rpm and heating to 80 °C to accelerate dissolution. The concentrated AgNPs colloid was then added to the PVA solution, and the resulting PVA-AgNPs mixture was used to fabricate nanocomposites by two methods: thin-film casting and electrospinning for fiber production.

### 2.4. Methods

Fourier-transform infrared (FTIR) spectroscopy was used to analyse the composition of the biological extracts. Spectra were recorded using a Bruker Tensor 27 spectrometer (Bruker, Billerica, MA, USA) (4000–400 cm^−1^, 64 scans, 4 cm^−1^ resolution). The samples were dried at 45 °C, mixed with KBr (1 mg sample: 200 mg KBr), and pressed into 13 mm pellets.

All prepared colloidal silver nanoparticle solutions were analysed using a UNICAM UV-vis spectrophotometer UV4 (Unicam Ltd., Cambridge, UK). Quartz semi-micro cuvettes with an optical path length of 10 mm were used, and the measurement range was set to 300–700 nm.

The size and morphology of the nanoparticles were examined using Transmission Electron Microscopy (TEM; JEOL model JEM-2000FX, acceleration voltage 200 kV, JEOL Ltd., Tokyo, Japan). For TEM sample preparation, a drop of the colloidal solution was deposited onto a copper grid coated with a carbon film, dried, and coated with an additional carbon layer to stabilize the nanoparticles. The presence of silver in the nanoparticles was confirmed by energy-dispersive X-ray spectroscopy (EDX) and selected area electron diffraction (SAED).

The presence and distribution of nanoparticles in the polymer composites (thin films and nanofibers) were further analysed using Scanning Electron Microscopy with a focused ion beam (SEM/FIB; ZEISS AURIGA Compact; ZEISS, Oberkochen, Germany).

Particle size and ζ-potential were measured using a ZetaSizer Nano ZS (Malvern, UK), equipped with a 4 mW He-Ne laser at a wavelength of 633 nm. Particle size measurements were carried out at a backscatter angle of 173°, while ζ-potential was measured using a DTS10710 capillary cuvette. Multiple repeated measurements were performed to ensure accuracy and reliability, with results processed automatically.

Composite PVA-AgNPs fibers were fabricated using electrospinning technology (Nanospider). During the electrospinning process, a voltage of 75 kV was applied, and the distance between the spinning and collecting electrodes was set to 120 mm.

### 2.5. Antibacterial Testing of AgNPs and Nanocomposites

The antibacterial activity of silver nanoparticles and prepared nanocomposites was evaluated against selected pathogenic microorganisms: *S. chromogenes*, *S. haemolyticus*, *S. aureus*, *S. uberis*, *E. coli*, and *P. aeruginosa*.

For colloidal AgNPs, the well diffusion method (WDM) was used. Petri dishes were prepared with Mueller–Hinton agar (MHA), and 100 μL of each bacterial suspension (adjusted to 0.5 McFarland standard, ≈1.5 × 10^8^ CFU/mL) was spread evenly across the surface. Wells (6 mm diameter) were punched into the agar, and 6 μL of test colloid was applied to each well. Negative controls included plant extract and deionized water, while positive controls consisted of silver nitrate solution (matching AgNPs precursor concentration) and gentamicin (ATB-GEN, 10 μg/mL). Plates were incubated at 37 °C for 24 h, and inhibition zones were measured in mm. All tests were performed in triplicate.

For nanocomposites, the disk diffusion method (DDM) was used. Circular samples (5 mm diameter) were cut from nanocomposite films and fibers and placed directly onto MHA plates previously inoculated with bacterial suspensions as described above. Pure PVA fibers and films (without AgNPs) served as negative controls. After incubation at 37 °C for 24 h, antibacterial activity was assessed by measuring the diameter of inhibition zones around each sample. Measurements were averaged by three independent replicates. The lavender leaf extract itself was also tested as a negative control under the same conditions and did not produce inhibition zones.

## 3. Results and Discussion

### 3.1. Silver Nanoparticles Analysis

#### 3.1.1. FTIR Spectroscopy of Precursor, Extract, and Colloid

**Figure 1:**
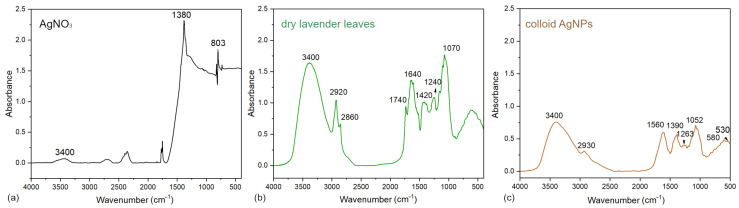
FTIR analysis of AgNO_3_ (**a**), leaf extract (**b**), and AgNPs colloid (**c**).

FTIR spectroscopy was used to identify functional groups presented in the silver precursor, lavender leaf extract, and the resulting AgNPs colloid, shown in Figure 1a.

The spectrum of AgNO_3_ showed a broad absorption band around 3400 cm^−1^, corresponding to O-H stretching vibrations, which may be attributed to hydrated nitrate. A sharp and intense band observed at 1380 cm^−1^ is characteristic of the symmetric stretching vibrations of the NO_3_^−^ ion. Additionally, a peak at 803 cm^−1^ is associated with the bending vibrations of the nitrate group [25,26,27,28], which consistently reports prominent NO_3_^−^ absorptions in the regions of ~1350–1385 cm^−1^ and ~800–830 cm^−1^.

The lavender leaves exhibited a complex FTIR spectrum Figure 1b, indicating a rich composition of bioactive compounds. A broad band at ~3400 cm^−1^ was attributed to O–H stretching vibrations of phenolic compounds and alcohols. Peaks at 2920 and 2860 cm^−1^ indicated C–H stretching of aliphatic chains. A strong band at 1740 cm^−1^ was assigned to C=O stretching vibrations from esters, aldehydes, or carboxylic acids. The band at 1640 cm^−1^ corresponds to C=C stretching or amide I vibrations, indicating the presence of proteins or aromatic compounds. Additional peaks at 1420 and 1240 cm^−1^ were assigned to C–H bending and C–O–C stretching, respectively, while the 1070 cm^−1^ band was attributed to C–O stretching of alcohols or polysaccharides. In conclusion, the bands associated with valence vibrations of –CH_2_, –OH, C=O, C=C, C–O, and –NH groups were observed, indicating the presence of phenols, flavonoids, proteins, and terpenoids [27].

Following the reduction of Ag^+^ ions and formation of AgNPs, the FTIR spectrum of the colloidal solution exhibited several changes Figure 1c. The O–H band at ~3400 cm^−1^ was retained, indicating the presence of hydroxyl-containing molecules on the nanoparticle surface. The C–H stretching bands were slightly shifted to 2930 cm^−1^. New or intensified bands appeared at 1560 cm^−1^ (amide II or C=O stretching), 1390 cm^−1^ (COO– or aromatic groups), 1263 and 1052 cm^−1^ (C–O–C or C–N stretching), and at 580 and 530 cm^−1^, which are indicative of metal–oxygen (Ag–O) interactions, confirming nanoparticle formation.

These spectral modifications confirm the involvement of phenolic compounds, flavonoids, and proteins in both the reduction and stabilization processes of AgNPs. A comparable FTIR-based interpretation was provided by Salayová et al. [24], who used lavender leaf extract for green synthesis of AgNPs and reported key absorption bands near 3400 cm^−1^ (O–H), 1634 cm^−1^ (C=O or amide I), 1372 cm^−1^ (aromatic C–N or COO^−^), and 1044 cm^−1^ (C–O stretching), consistent with phenolic and proteinaceous capping agents. Despite slight shifts in band positions, likely due to variations in extraction protocols or plant chemotypes, their findings confirm that lavender metabolites effectively mediate silver ion reduction and nanoparticle stabilization [28]. The disappearance or weakening of nitrate-related peaks at 1380 and 803 cm^−1^ further indicated efficient conversion of Ag^+^ to Ag^0^, consistent with similar FTIR observations by Abbas et al. [25], Hezma et al. [26], and Kora et al. [27].

Collectively, these results demonstrate that the lavender leaf extract acts as both a reducing and stabilizing system during nanoparticle formation. Its phenolic compounds, flavonoids, proteins, and other bioactive metabolites facilitate electron transfer from functional groups (–OH, C=O, C=C, –NH) to silver ions, leading to the formation of metallic Ag^0^. The same functional moieties remain adsorbed on the nanoparticle surface, forming an organic capping layer that ensures colloidal stability. This dual mechanism of reduction and surface passivation is characteristic of plant-mediated synthesis and underscores the multifunctional role of phytochemicals in controlling nanoparticle formation and stabilization.

#### 3.1.2. Mechanism of AgNPs Formation and Stabilization

The formation of AgNPs mediated by *Lavandula angustifolia* leaf extract can be explained by a typical plant-assisted (green) mechanism involving the reduction of Ag^+^ ions, nucleation and growth of metallic Ag^0^, and surface passivation by phytochemicals acting as capping agents. Plant extracts contain diverse metabolites such as phenolic acids, flavonoids, terpenoids, reducing sugars, and proteins, which may serve as electron donors and contribute to nanoparticle stabilization. During the reaction, these compounds reduce Ag^+^ from AgNO_3_ to Ag^0^, initiating the nucleation and subsequent growth of nanoparticles. Meanwhile, a fraction of the biomolecules remains adsorbed on the Ag surface, creating an organic shell that limits aggregation and enhances colloidal stability. This dual role of plant metabolites (reduction and stabilization) has been widely discussed for plant-mediated AgNPs synthesis.

FTIR spectra support the involvement of hydroxyl and carbonyl-containing compounds, as well as amide-related bands, indicating that polyphenols and/or proteinaceous molecules participate in the process and remain associated with the nanoparticle surface. The retention/shift in bands attributed to –OH, C=O, and –NH groups after nanoparticle formation is consistent with surface binding of phytochemicals and steric stabilization. Moreover, the long-term stability observed in UV-Vis monitoring, together with the ζ-potential of −15.9 mV, suggests that electrostatic stabilization is moderate and that steric effects provided by the organic capping layer likely play a dominant role in preventing aggregation [28,29,30].

#### 3.1.3. UV-vis Analysis

**Figure 2:**
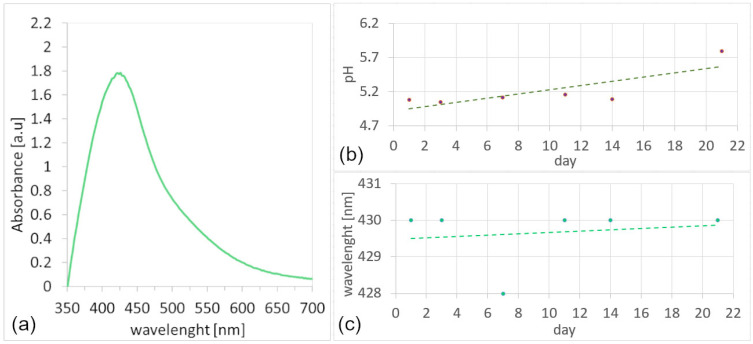
UV-vis spectrum of the colloidal AgNPs solution (**a**), pH variation in the colloid over 21 days (**b**), and stability of the SPR peak wavelength during storage (**c**).

UV-vis spectroscopy was employed to confirm the formation of AgNPs synthesized using lavender leaf extract and to assess the reducing capability of the extract. Upon mixing the extract with the silver precursor, a rapid color change to yellow brown indicated the onset of nanoparticle formation [31]. The UV-vis spectrum recorded on the first day (D1) after synthesis, shown in Figure 2a, displayed a distinct surface plasmon resonance (SPR) band, confirming AgNPs formation.

The main spectral parameters, such as absorbance intensity (ABS_max_), spectral symmetry, and the wavelength of maximum absorbance (λ_max_), were used to evaluate nanoparticle properties [32]. The obtained spectrum showed a strong and symmetric SPR peak at 424 nm with ABS_max_ = 1.80, corresponding to uniform, predominantly spherical nanoparticles [33,34,35]. The stability of the colloidal solution was further verified over 21 days of storage, during which no significant spectral shift or intensity loss was observed, indicating excellent dispersion stability of the lavender-mediated AgNPs, Figure 2c.

The colloidal solution pH, shown in Figure 2b, remained relatively stable during the 21-day monitoring period, showing only a slight gradual increase from approximately 5.1 (D1) to 5.7 (D21). Although a minor deviation was observed on the final day, it did not significantly alter the overall trend of the fitted line, indicating that no substantial pH drift occurred. Such minor changes can be attributed to slow chemical transformations in the system; however, they had no adverse effect on nanoparticle stability.

Similarly, the position of the SPR absorption maximum (λ_max_) remained nearly constant, within the range of 429–430 nm, confirming the absence of significant nanoparticle growth or aggregation during storage. These findings demonstrate that the lavender leaf extract exhibits excellent reducing and stabilizing efficiency, producing a colloid with high long-term stability and practical suitability for storage.

#### 3.1.4. Characterization of AgNPs by TEM, HRTEM, EDX, DLS, and Zeta Potential

**Figure 3:**
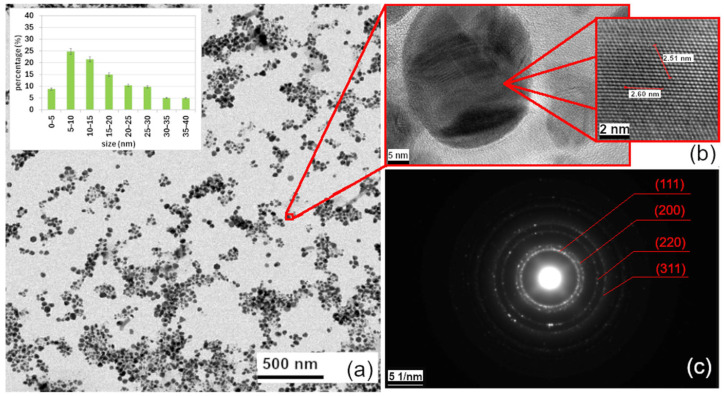
TEM image of AgNPs (inner picture shows size distribution histogram of AgNPs) (**a**), detailed view of a nanoparticle (**b**), and SAED pattern of the sample (**c**).

TEM imaging confirmed the assumption based on the UV-vis analysis, which suggested the formation of silver nanoparticles with a predominantly spherical shape, shown in Figure 3a. The particle size distribution of AgNPs was determined based on TEM image analysis. As shown in Figure 3a inner picture, the AgNPs exhibited a size range from 2 to 40 nm, with most particles falling within the 5–20 nm interval (~70%). The average particle diameter was calculated to be 16.2 ± 7.3 nm, confirming the formation of quasi-spherical nanoparticles with a relatively narrow size distribution.

Figure 3b shows a detailed view of one of the nanoparticles, with interatomic distance measurements from HRTEM (2.51 nm and 2.60 nm, measured across 10 atoms in two different directions). These measurements confirm the presence of silver. An electron diffraction (SAED), shown in Figure 3c, also supports the presence of silver, with the obtained interplanar spacings (d) in agreement with values from the JCPDS-ICDD database, card no. 4-783. The crystallographic planes (111), (200), (220), and (311), along with the experimentally measured d-values for these planes (2.359 Å, 2.044 Å, 1.445 Å, and 1.231 Å), are consistent with results reported by others [36,37]. These findings confirm both the morphology and crystalline structure of the synthesized AgNPs.

**Figure 4:**
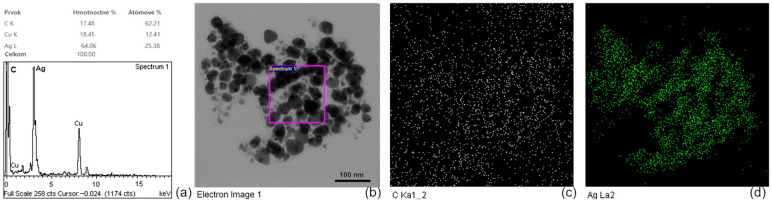
EDX analysis of AgNPs (**a**); TEM image of AgNPs with EDX acquisition area (**b**); elemental mapping of carbon (**c**); and elemental mapping of silver (**d**).

The EDX spectrum, shown in Figure 4a, confirms the elemental composition of the sample, with distinct peaks corresponding to silver. The TEM image, shown in Figure 4b, shows spherical AgNPs with uniform size distribution. The purple square marks the area used for EDX acquisition. Elemental mapping confirms the presence and spatial distribution of carbon, shown in Figure 4c, related to the sample preparation, and silver, shown in Figure 4d, demonstrating a high density of AgNPs across the field of view.

To further assess particle size distribution in suspension and evaluate colloidal stability, dynamic light scattering (DLS) analysis was performed.

**Figure 5:**
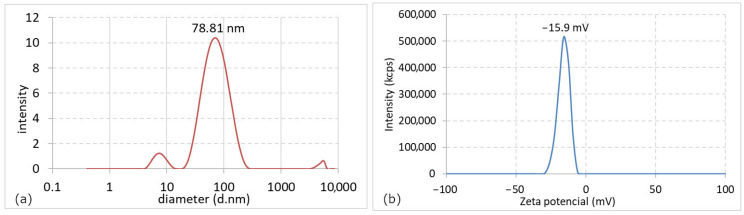
Particle size distribution determined by DLS (**a**) and zeta potential (**b**).

Figure 5a presents the results of DLS analysis of the AgNPs colloid. The dominant particle diameter was approximately 78.81 nm, representing 93% of the total sample volume, while a secondary peak at around 7.53 nm (6%) likely corresponds to smaller nanoparticles or residual clusters. These results differ from TEM observations, which revealed predominantly spherical nanoparticles with an average core diameter of ~20 nm. This discrepancy arises from the fundamental differences between the two techniques: DLS measures the hydrodynamic diameter of nanoparticles in suspension, which includes the solvation layer and any adsorbed biomolecules, whereas TEM provides direct visualization of the metallic cores. The larger apparent size obtained from DLS thus can suggest that proteins and other organic compounds from the lavender extract were adsorbed onto the AgNPs surface, forming a stabilizing layer that enhances colloidal stability.

This interpretation is consistent with the FTIR analysis, which confirmed the presence of functional groups such as –OH, C=O, and –NH, indicating that phenolic compounds and proteins participated in the reduction of Ag^+^ ions and remained bound to the nanoparticle surface, contributing to their effective stabilization.

The polydispersity index (PdI) obtained from DLS was 0.331, indicating a moderately polydisperse colloidal system.

Figure 5b shows the ζ-potential distribution of the analysed colloid, with a peak value of −15.9 mV, indicating a slightly negative surface charge. The ζ-potential is a key parameter reflecting the electrostatic stability of colloidal systems. Values above ±30 mV typically denote strong electrostatic repulsion and high stability, whereas lower values suggest limited electrostatic contribution to stabilization. In this case, the measured ζ-potential indicates moderate electrostatic stability, which is consistent with literature reports for biologically synthesized AgNPs that typically exhibit ζ-potential values in the range of approximately −10 to −25 mV [38].

However, considering the long-term stability observed in UV-vis monitoring and the presence of biomolecules adsorbed on the nanoparticle surface, it can be concluded that steric stabilization provided by proteins and phenolic compounds from the lavender extract plays a dominant role. Therefore, additional surface charge modification or the use of external stabilizing agents was not necessary in this system.

#### 3.1.5. Antibacterial Activity of Colloidal AgNPs

To extend the practical applicability of the synthesized AgNPs, their antimicrobial properties were evaluated by the well diffusion method against selected microorganisms, including Gram-positive (*S. aureus*, *S. haemolyticus*, *Str. uberis*) and Gram-negative (*E. coli*, *P. aeruginosa*) strains. In all cases, clear inhibition zones (IZ) were observed, confirming the antibacterial efficacy of the biogenic AgNPs. The inhibition zone diameters ranged from 7.99 to 9.68 mm, with the highest activity recorded for *S. haemolyticus* (9.68 mm) and *E. coli* (9.56 mm), while *P. aeruginosa* showed the lowest susceptibility (7.99 mm). Similar broad-spectrum antibacterial performance of green-synthesized AgNPs has been reported in the literature [39,40]. Importantly, the plant extract alone showed no inhibition zones, confirming that the antibacterial effect originated from AgNPs.

Given these results, the AgNPs were subsequently incorporated into a polyvinyl alcohol (PVA) matrix to evaluate whether the antibacterial activity is retained after embedding into the polymer and to compare the performance of the resulting nanocomposites in different material forms (fibers and films).

### 3.2. Analysis of Nanocomposites

**Figure 6:**
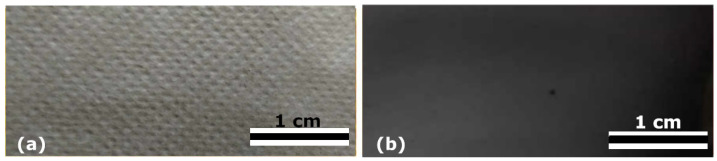
AgNPsLV nonwoven textile (**a**), AgNPsLF thin foil (**b**).

Polyvinyl alcohol (PVA) is a water-soluble, biocompatible, and low-toxic polymer commonly used as a matrix for nanocomposites due to its excellent film-forming and mechanical properties. In this study, prepared silver nanoparticles were incorporated into the PVA matrix via the ex situ method. The process resulted in nanocomposite materials in the form of nonwoven textile mats, Figure 6a, and thin polymer films, Figure 6b, denoted as AgNPsLV and AgNPsLF, respectively.

The incorporation of metal nanoparticles into polymer matrices is strongly influenced by the physicochemical properties of both components and by the processing conditions applied during composite fabrication. Achieving uniform nanoparticle dispersion is crucial, as aggregation can significantly affect the resulting materials’ mechanical, optical, and biological performance.

Several studies have emphasized the positive role of ultrasonic treatment in breaking nanoparticle agglomerates, either directly in the colloid before incorporation or within the polymer–nanoparticle mixture before fiber formation, thereby improving homogeneity [32,34]. In research it is often demonstrated that optimized ultrasound conditions ensured significantly better particle distribution compared to mechanical stirring. Similarly, Kumar et al. [41] reported that in situ incorporation of AgNPs led to uniform distribution and enhanced mechanical strength, while Gogoi et al. [42] showed that the ex situ method offers greater flexibility, enabling controlled AgNPs release and improved catalytic and antibacterial performance. Other authors [43] confirmed that ex situ incorporation into biopolymer matrices, such as starch or cellulose, enhances optical properties, particularly when the nanoparticle concentration is optimized.

Not only the homogeneous distribution of nanoparticles in the polymer, but also their surface presence is important, especially regarding the toxicity of such composites. According to Shetty et al. [44], nanoparticles located on the fiber surface tend to become trapped within surface pores and are maintained primarily by electrostatic interactions with the polymer chains. The authors observed that increased nanoparticle concentration or enhanced homogenization could improve uniformity within nanofibers and thin films. Moreover, their presence on the surface may contribute to direct interaction with the surrounding environment and pathogenic cells, thereby likely increasing antibacterial properties.

Considering these findings, several experimental approaches were tested in our study to determine the optimal procedure for incorporating biogenic AgNPs into the PVA matrix. The conditions varied mainly in polymer concentration, nanoparticle content, ultrasonic treatment, and electrospinning parameters.

During the first experiments, a series of modifications was performed to improve nanoparticle dispersion. These included adjustments to PVA concentration (6–8%), colloid concentration (50–500 mg/L), mixing and electrospinning parameters. However, SEM analysis revealed that the resulting nanocomposites exhibited non-uniform nanoparticle distribution and aggregation. Even with increased nanoparticle concentration or longer homogenization time, the dispersion remained unsatisfactory, indicating that the processing parameters were not yet optimal for achieving homogeneous embedding of AgNPs.

Based on these results, an optimized preparation was established. For the optimized, 47 mL of the AgNPs colloid (c_Ag_ = 500 mg/L) was concentrated by centrifugation (9000 rpm, 20 min), and the sediment was subsequently redispersed and sonicated to disrupt possible agglomerates. Polyvinyl alcohol (PVA, 6 wt.%) was prepared by dissolving the polymer in deionized water at 80 °C under continuous stirring (600 rpm) for 60 min. The concentrated and sonicated AgNPs dispersion was then added to the PVA solution, and the resulting mixture was further sonicated for 15 min to ensure homogeneous nanoparticle distribution before processing. Nanocomposite nanofibers were fabricated by electrospinning using a Nanospider at a voltage of 75 kV and an electrode distance of 120 mm. Thin films were prepared from the same dispersion by casting followed by drying under ambient conditions.

**Figure 7:**
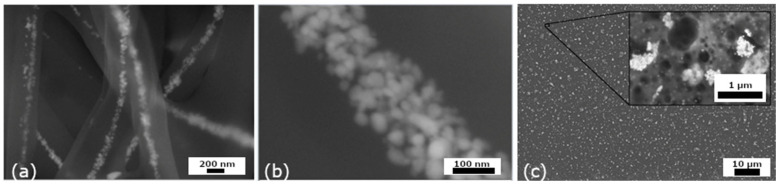
SEM micrograph of AgNPsLV500-47 fibers (**a**), detail of fibers (**b**), and AgNPsLF500-47 thin foil with detail (**c**).

This optimized approach produced uniform nanofibers with diameters ranging from 200 to 500 nm, containing evenly distributed AgNPs along their length, shown in Figure 7a,b. Only minor localized agglomerates were observed. Importantly, the ex situ incorporation process did not affect the morphology or size of the nanoparticles. Such uniform dispersion and structural integrity are essential for ensuring the reproducibility and stability of the resulting nanocomposite material.

The SEM micrograph of the thin polymer film, shown in Figure 7c, revealed a relatively homogeneous distribution of AgNPs across the film matrix, accompanied by limited surface clustering. Most nanoparticles were incorporated within the film structure, with a smaller fraction visible on the surface, detailed in Figure 7c, which may contribute to the material’s surface reactivity and antibacterial efficiency.

Considering the homogeneity, stability, and visible uniformity of the nanocomposite prepared under these optimized conditions, this material was selected for subsequent biological testing. The incorporation of AgNPs into the PVA matrix was optimized with the aim of achieving the most homogeneous nanoparticle distribution within the polymer. At a silver nanoparticle content of 0.8 wt.%, a uniform dispersion of AgNPs throughout the matrix was obtained.

### 3.3. Antibacterial Activity of Nanocomposites

Antibacterial tests were performed on nanocomposites, electrospun fibers (AgNPsLV), and cast films (AgNPsLF), containing green-synthesized silver nanoparticles. Two major bacterial groups were evaluated: Gram-positive (*S. aureus*, *S. haemolyticus*, *S. uberis*) and Gram-negative (*E. coli*, *P. aeruginosa*). Pure PVA fibers served as the negative control.

**Figure 8:**
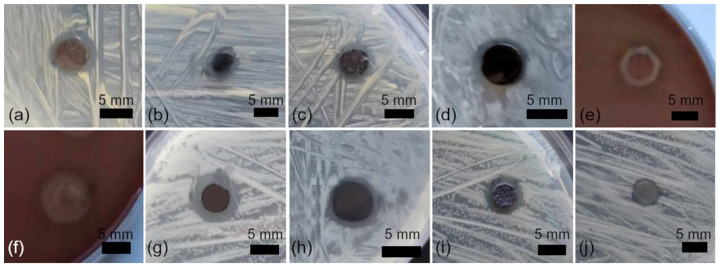
Antibacterial activity of polymer nanocomposites for *S. aureus*—fibers (**a**), foil (**b**); *S. haemolyticus*—fibers (**c**), foil (**d**); *S. uberis*—fibers (**e**), foil (**f**); *E. coli*—fibers (**g**), foil (**h**); *P. aeruginosa*—fibers (**i**), foil (**j**).

**Figure 9:**
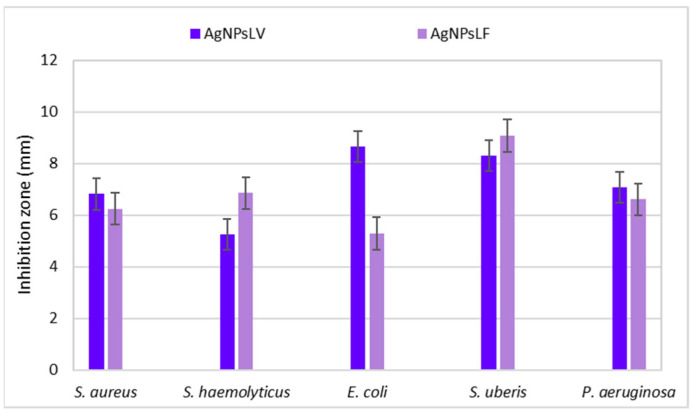
Antibacterial activity of polymer nanocomposites (mean ± SD, *n* = 3).

The antibacterial activity of both nanocomposite forms, shown in Figure 8, was quantified by measuring inhibition zone diameters, Figure 9. All PVA-AgNPs samples produced distinct inhibition zones against both Gram-positive and Gram-negative strains, while pure PVA controls exhibited no inhibition zones (0 mm; Supplementary Material). For electrospun fibers, inhibition zone diameters ranged from 5.256 to 8.666 mm, with the strongest effects observed against *E. coli* (8.666 mm) and *S. uberis* (8.305 mm). For thin films, inhibition zones ranged from 5.299 to 9.076 mm, with the highest activity recorded against *S. uberis* (9.076 mm) and *S. haemolyticus* (6.856 mm). Overall, Gram-positive strains tended to exhibit slightly higher sensitivity in several cases.

Differences in the interaction of nanocomposites with Gram-positive and Gram-negative bacteria are related to structural variations in their cell envelopes. Gram-negative bacteria possess an additional outer membrane rich in polysaccharides and lipoproteins, which acts as a barrier limiting the penetration of metal ions and nanoparticulate species. In addition, active efflux mechanisms may contribute to reduced intracellular accumulation of antimicrobial agents. Gram-positive bacteria, in contrast, lack this outer membrane, which may facilitate interactions with silver-containing nanocomposites [10,45].

This test also showed that there is no significant difference between the effectiveness of fibers and films, even though it could be assumed that nonwoven fabrics, due to the higher proportion of surface capable of direct contact with the environment, should exhibit more pronounced antibacterial properties. In PVA-AgNPs nanocomposites, nanofibrous structures have been reported to exhibit enhanced antibacterial activity compared to films due to their higher specific surface area and porosity, which facilitate contact-mediated antimicrobial action and the release of Ag^+^ species [19,37]. In the present study, however, the difference between fibers and films was not uniform across all tested strains: fibers were more effective against *E. coli* (8.666 vs. 5.299 mm) and slightly against *S. aureus* (6.824 vs. 6.255 mm), whereas films performed better against *S. haemolyticus* (6.856 vs. 5.256 mm) and *S. uberis* (9.076 vs. 8.305 mm). This indicates that the antibacterial response is strain-dependent and may be influenced by nanoparticle accessibility at the material surface, local AgNPs distribution within the polymer matrix, and differences in bacterial cell-envelope structure and permeability [10,41]. Similar PVA-based AgNPs nanocomposites reported in the literature exhibit measurable inhibition zones and broad-spectrum antibacterial activity, supporting the suitability of PVA as a carrier enabling effective antimicrobial performance of incorporated AgNPs.

Yontar and Çevik [46] prepared PVA nanocomposite films containing green-synthesized AgNPs incorporated ex situ into the polymer matrix. Their antibacterial assays, including growth curves and inhibition zone measurements, demonstrated significant inhibition of *E. coli* and *S. aureus*, with antibacterial efficacy increasing with increasing AgNPs content.

Ragab et al. [47] fabricated PVA/chitosan hydrogel films reinforced with green-synthesized AgNPs obtained using plant extracts (Aloe vera and green tea) as reducing and stabilizing agents. The hydrogels exhibited good antibacterial activity against *E. coli* and *S. aureus* while maintaining high cell viability (~82%), indicating good biocompatibility and suitability for wound healing applications.

Shehzad et al. [48] reported PVA hydrogels containing green-synthesized AgNPs prepared using *Papaver somniferum* extract. Uniformly dispersed AgNPs with an average size of approximately 10 nm were obtained, and pronounced antibacterial activity was observed, with reported inhibition zones of approximately 13.5 mm for *S. aureus*, 14 mm for *Klebsiella pneumoniae*, 13 mm for *Proteus mirabilis*, and 12.5 mm for *P. aeruginosa*.

In comparison with these studies, the inhibition zone diameters observed in the present work are generally lower. This can be attributed to differences in nanoparticle concentration, polymer matrix composition, incorporation strategy (ex situ versus in situ), and nanoparticle distribution within the matrix. These factors critically influence silver ion release, nanoparticle accessibility at the material surface, and thus the overall antibacterial performance. Consequently, variations in inhibition zone values among different studies are expected and reflect system-specific characteristics rather than inconsistencies in antibacterial efficacy.

## 4. Conclusions

Silver nanoparticles were successfully synthesized using lavender leaf extract, yielding predominantly spherical particles with an average diameter of 16 nm. The synthesized AgNPs exhibited clear antibacterial activity, confirming their biological functionality. Subsequently, the nanoparticles were incorporated into a PVA polymer matrix using an ex situ method. Various incorporation ratios and processing parameters were tested, and the optimized procedure resulted in a homogeneous nanocomposite with preserved nanoparticle morphology and stable antimicrobial performance.

The study demonstrated the feasibility of preparing functional PVA-AgNPs nanocomposites with enhanced antibacterial properties, confirming the successful transfer of AgNPs functionality into the polymer system. These materials represent a promising basis for further development of antimicrobial coatings, films, and packaging materials with potential applications in biomedical and environmental fields.

## Data Availability

The original contributions presented in the study are included in the article; further inquiries can be directed to the corresponding author.

## Supplementary Materials

The following supporting information can be downloaded at: https://www.mdpi.com/article/10.3390/nano16020098/s1, Figure S1: Results of the toxicity test against *S. uberis* for fibers (a) and films (b), *E. coli* for fibers (c) and films (d), *S. aureus* for fibers (e) and films (f), and *P. aeruginosa* for fibers (g) and films (h); Figure S2: Biological activity of the lavender leaf extract evaluated on *P. aeruginosa* (left) and on the algae *Ch. kessleri* (right).
