# Supplementary material for: Sustainable Lavender Extract-Mediated Synthesis of Silver Nanoparticles and Their Use in Fabricating Antibacterial Polymer Nanocomposites
Source: Nanomaterials (Basel). 2026 Jan 12;16(2):98. doi: 10.3390/nano16020098 (PMC12844991; doi:10.3390/nano16020098)
Supplement: Supplementary file 1 [file nanomaterials-16-00098-s001.zip › nanomaterials-4074165-supplementary.pdf]

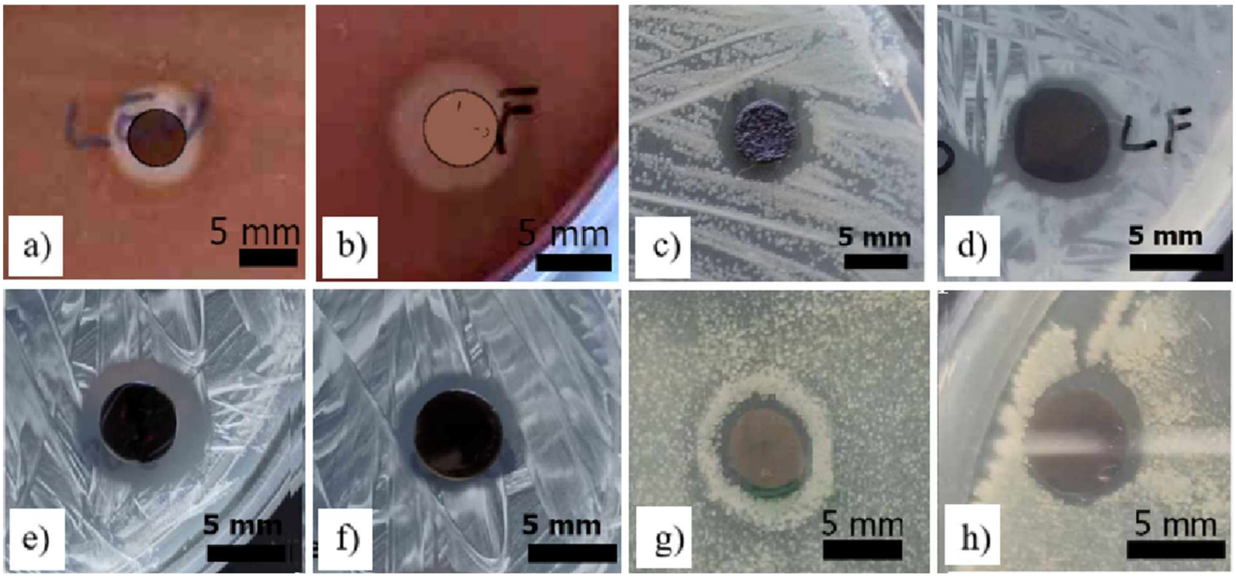

Figure S1 Results of the toxicity test against S. uberis for fibers (a) and films (b), E. coli for fibers (c) and films (d), S. aureus for fibers (e) and films (f), and P. aeruginosa for fibers (g) and films (h)

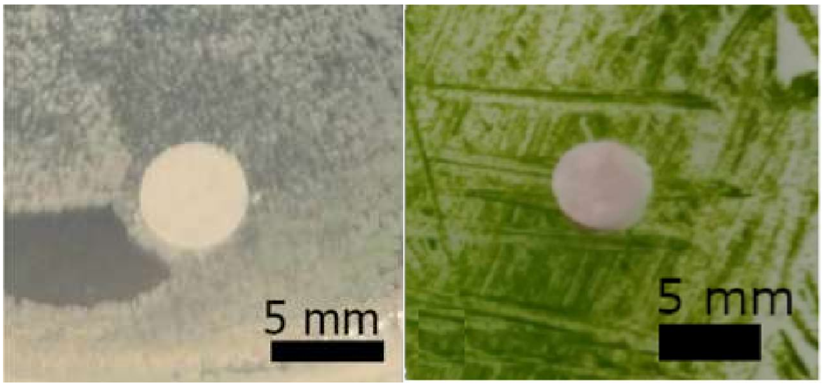

Figure S2 Biological activity of the lavender leaf extract evaluated on *P. aeruginosa* (left) and on the algae *Ch. kessleri* (right).
